# A rodent model of human dose-equivalent progestin-only implantable contraception

**DOI:** 10.1186/s12958-021-00729-w

**Published:** 2021-03-22

**Authors:** Heather C. M. Allaway, Roger A. Pierson, Jesse Invik, Susan A. Bloomfield

**Affiliations:** 1 Department of Health and Kinesiology, Texas A&M University, College Station, TX USA; 2 Department of Obstetrics & Gynecology, University of Saskatchewan, Saskatoon, SK Canada; 3 Synergyne Imaging Technology Inc, Saskatoon, SK Canada

**Keywords:** Hormonal contraception, Long-acting reversible contraception, Progestin, Etonogestrel, Estrus cycle, Ovarian function, Ultrasound biomicroscopy

## Abstract

**Background:**

Long-acting, reversible contraceptives (LARC; progestin only) are an increasingly common hormonal contraceptive choice in reproductive aged women looking to suppress ovarian function and menstrual cyclicity. The overall objective was to develop and validate a rodent model of implanted etonogestrel (ENG) LARC, at body size equivalent doses to the average dose received by women during each of the first 3 years of ENG subdermal rod LARC use.

**Methods:**

Intact, virgin, female Sprague-Dawley rats (16-wk-old) were randomized to 1 of 4 groups (*n* = 8/group) of ENG LARC (high-0.30μg/d, medium-0.17μg/d, low-0.09μg/d, placebo-0.00μg/d) via a slow-release pellet implanted subcutaneously. Animals were monitored for 21 days before and 29 days following pellet implantation using vaginal smears, ultrasound biomicroscopy (UBM), saphenous blood draws, food consumption, and body weights. Data were analyzed by chi-square, non-parametric, univariate, and repeated measures 2-way ANOVA.

**Results:**

Prior to pellet implantation there was no difference in time spent in estrus cycle phases among the treatment groups (*p* > 0.30). Following pellet implantation there was a dose-dependent impact on the time spent in diestrus and estrus (*p* < 0.05), with the high dose group spending more days in diestrus and fewer days in estrus. Prior to pellet insertion there was not an association between treatment group and estrus cycle classification (*p* = 0.57) but following pellet implantation there was a dose-dependent association with cycle classification (*p* < 0.02). Measurements from the UBM (ovarian volume, follicle count, corpora lutea count) indicate an alteration of ovarian function following pellet implantation.

**Conclusion:**

Assessment of estrus cyclicity indicated a dose-response relationship in the shift to a larger number of acyclic rats and longer in duration spent in the diestrus phase. Therefore, each dose in this model mimics some of the changes observed in the ovaries of women using ENG LARC and provides an opportunity for investigating the impacts on non-reproductive tissues in the future.

## Background

Hormonal contraception (HC) is routinely used by reproductive age women (18–49 years old) to suppress ovarian function and menstrual cycling. The HC modality is a personal choice based on knowledge, preference/comfort level, prescription coverage, and discussions with an individuals’ health care provider. Of the 72.2 million women accounted for in the National Center for Health Statistics December 2018 updated Data Brief on contraception use, 26% were using HC [1]. Of the 18.8 million women using HC, 40% were using long-acting, reversible contraception (LARC); women 20–39 years old represent the largest proportion of LARC users [1].

There are three modalities of progestin-only LARC: intrauterine device (IUD), subdermal rods, and injectable. Current available IUD options secrete levonogestrel (LNG) and the subdermal rod options secrete etonogestrel (ENG). Due to differences in implantation location (uterus vs. arm), the IUD releases LNG at rates of 14-20μg/d initially [2], while the subdermal rods release ENG at 70μg/d initially [3, 4]. The rate of release and dose slowly decrease over the 3 or more years of use for both types of implanted LARC. The injectable LARC uses depot-medroxyprogesterone acetate (150 mg every 3 months) and is associated with a decrease in bone mineral density (BMD) [5, 6], likely caused by induction of the severe hypoestrogenism and amenorrhea [6–9]. Subdermal rod and IUD LARC modalities provide multiple advantages to women, including a high efficacy independent of patient compliance, long term unattended use (3 or more years), highly effective ovarian and menstrual suppression [10] and reduced risk for venous thromboembolism and breast cancer compared with combined oral contraception use [11]. Unlike injectable LARC, approximately 29% of subdermal rod and IUD LARC users achieve full suppression of ovulation and amenorrhea without inducing severe hypoestrogenism [10, 12, 13]. For subdermal rod and IUD LARC modalities the secretion of progestin decreases slowly over time [12], while remaining well above the contraceptive efficacy threshold. Estradiol concentrations decrease initially to early follicular phase concentrations, but rise gradually over the years of implantation [12], coinciding with the gradual decrease in progestin release, and vary with the growth of ovarian follicles over time. Adequate estrogen exposure is important in neurological, cardiovascular, and musculoskeletal protection, atrial vasodilation, healthy immunity and liver protein function, as well as breast and endometrial tissue health [14, 15].

The use of IUD and subdermal rod LARC modalities as contraceptive methods are increasing worldwide due to the ease of compliance, long duration of use, high efficacy, few contraindications, and stable progesterone concentrations in serum [10]. Despite the widespread and long-term use of HC, medical and scientific community have failed to determine whether HC therapy use is helpful or harmful to non-reproductive body systems in the millions of women who use them worldwide. The use of HC to reduce the risk of unwanted pregnancy must be weighed against the incompletely defined risk for adverse effects on non-reproductive organ systems. Thus, it is important to understand the physiologic impact of subdermal rod and IUD LARC modalities on non-reproductive organ systems to facilitate improved best practices of HC prescription.

Rodents are long standing animal models used to assess reproductive health-related pharmaceutical interventions due to the similarities of relationships among the hypothalamic, pituitary, ovarian axis of the rat and human [16]. Studies on implantable LARC suppression of reproductive function in rodents have used the subdermal rods (Implanon, Organon, Oss, Netherlands) cut to 5 μm thick [17–19]. Animals dosed using this methodology demonstrated suppressed ovarian function; however, the circulating concentrations of progestin and estradiol were not evaluated [17–19]. Animals received a supraphysiologic dose of progestin in the afore mentioned studies [17–19] due to the properties of the ethylene vinyl acetate matrix in which the ENG is dispersed. Supraphysiologic doses of progesterone suppresses differentiation of osteoblast cells in culture [20]; therefore, using this type of dosing scheme would not result in translational mechanistic effects in all non-reproductive organ systems.

Development of an animal model with LARC dosing physiologically equivalent to that used by women over the usual 3 years of use will enable future research exploring more mechanisms for the physiologic impact of implantable LARC, including that on non-reproductive organ systems. The objective of the present study was to develop and validate a rodent model of implanted LARC using ENG doses at physiologic equivalents to the average dose received by women during each of the first 3 years of use. We hypothesized there would be a dose-dependent suppression of estrus cyclicity, with the high dose having the greatest suppressive effect corroborated by measures of ovarian function (ultrasound biomicroscopy and serum estradiol).

## Materials and methods

### Animals and study design

Thirty-two virgin, female Sprague-Dawley rats (16-weeks-old; weight range 227-293 g) were purchased from Envigo Laboratories (Houston, TX) and allowed to acclimate to their surroundings, single housing, and diet (Research Diets D10012G, Research Diets, Inc., New Brunswick, NJ) for 2 weeks. At 16 weeks of age, female Sprague-Dawley rats are both reproductively and skeletally mature. Virgin animals were used to reduce inter-animal variability. Animals were housed in a temperature-controlled (23 ± 2 °C) room with a 12-h light-dark cycle in an AAALAC-accredited animal care facility. Animals had ad libitum access to food and water. Biweekly body weights and daily food intakes were assessed throughout the study.

Daily vaginal smear assessment of estrus cycling began day 1 of week 2 and every-other-day ultrasound biomicroscopy assessment of ovarian function began day 1 of week 3. Animals were block randomized on day 1 of week 4 based on body weight to placebo or one of three doses of ENG LARC (*n* = 8/group) delivered by slow-release pellets implanted at randomization. ENG pellet implantation and ultrasound procedures were performed under light isofluorane anesthesia (≤2%). Animal total study exposure to isofluorane did not differ among the treatment groups (range 190–330 min; *p* = 0.694). LARC pellets remained in place for 28–29 days, with weekly collections of saphenous vein serum samples. Following the LARC exposure period, animals were anesthetized via intraperitoneal injection of ketamine and dexmedetomidine (a ratio of 3:2; Henry Schein Animal Health, Dublin, OH, USA) and euthanized via exsanguination and decapitation. All animal procedures were performed in accordance with the National Institutes of Health guidelines for the humane care of laboratory animals and were approved by the Texas A&M University Institutional Animals Care and Use Committee.

### Estrus cycle monitoring

Estrus stage was determined from vaginal smears collected every morning between 0800 and 0830 h. Smears obtained with a cotton swab were placed on a slide and later examined at 400x with light microscopy (Cole Parmer, Vernon, Illinois) and stage of the estrus cycle was estimated based on predominant cell type observed in multiple fields of view [16, 21]. The estrus cycle has four histologically defined stages: proestrus (round nucleated epithelial cells), estrus (enucleated cornified cells), metestrus (proportional numbers of leukocytes and cornified cells), and diestrus (few cells, predominantly leukocytes, with the presence of thick mucus). Smears were obtained over a 43-day period (14 days pre-pellet insertion and 28 days post-pellet insertion). For assessment of cycle classification, the post-pellet insertion period was broken into two-week periods of time (early and late post-insertion). Rats that persisted in any one stage for 7 days or longer were considered acyclic and were considered irregular if estrus cycles were 7 days or longer.

### Etonogestrel (ENG) dosing and LARC implantation

Calculations of ENG dosing were based on physiologic dosing calculations for norgestrel by Igunnu et al. [22]. Our ENG release rates were calculated to be equivalent to the release rate for the human ENG subdermal rod LARC modality at years 1 (70 μg/d), 2 (40 μg/d), and 3 (20 μg/d) of use based on per kg body weight of the average North American woman [3, 13, 23]. The calculated ENG release rates for our three ENG doses were: 0.30 μg/d (High, as during year 1), 0.17 μg/d (Medium, as during year 2) and 0.09 μg/d (Low, as during year 3). The placebo group had a pellet of the biodegradable carrier-binder alone inserted (Innovative Research of America, Sarasota, FL). Once anesthetized, each rat was positioned in sternal recumbency and maintained on continuous 1–2% isofluorane. A 2 × 2 cm patch of fur was removed from the middle of the upper back between the shoulder blades. A 1 cm cranial-caudal incision was made between the shoulder blades and forceps were used to blunt dissect the skin from the muscle, creating a pocket between the ear and the shoulder. The forceps were then used to place the pellet into the pocket. A sterile suture staple was used to close the incision between the shoulder blades. Animals were monitored carefully during the hours following pellet insertion and all incisions were healed within 5 days.

### Ultrasound biomicroscopy

Ovarian follicular development was assessed by transabdominal ultrasound biomicroscopy based on published scanning techniques [24, 25]. The ultrasound biomicroscope used (UBM; Vevo 3100, Visual Sonics, Toronto, Canada) was a high-resolution acoustic imaging system consisting of a mechanical scan-head and associated signal and image processing hardware. A linear-array transducer (MX550D) with a center transmit frequency of 40 MHz, axial resolution of 40 μm, and field of view 15 mm × 11 mm was utilized. The maximum imaging depth was ~ 12 mm at the 40 MHz center frequency used. A focal point was maintained between 4 to 7 mm from the transducer surface. All scanning occurred between 0830 and 1200 h to minimize diurnal variation and scan order was randomized weekly. Scans were obtained every-other-day over a 35-day period (7 days pre-pellet insertion and 28–29 days post-pellet insertion). For change over time assessment, the post-pellet insertion period was broken into two week periods of time (early and late post-insertion).

Once anesthetized, each rat was positioned in dorsal recumbency, placed on a heating pad and maintained on continuous 1–2% isofluorane. The fore- and hind limbs were restrained with self-adherent elastic wrap to stretch the abdominal skin tight and the ventrolateral body hair coat was removed by shaving with a beard trimmer (Model 9918C, Wahl Clipper Corp., Sterling, IL, USA), then further cleared with sensitive skin hair remover cream (NAIR, Church & Dwight Canada Corp., Mississauga, ON, Canada) as needed. Ultrasound contact gel (EcoGel, Eco-Med Pharmaceuticals, Etobicoke, ON, Canada) was applied to the hair-free aspects of both the left and right side of the lateral abdomen. The transducer was positioned manually (all scans performed by HCMA) on the right dorsolateral side of the rat and moved cranially to caudally until the right ovary was visualized. The kidneys and liver were used as landmarks. Once the ovary was identified, the ovary was manually scanned medial to lateral and a cine-loop was recorded digitally (700 frames/10 s). The same procedure was followed on the left dorsolateral side to image the left ovary.

### Counting and measuring of follicles and corpora lutea

**Fig. 1:**
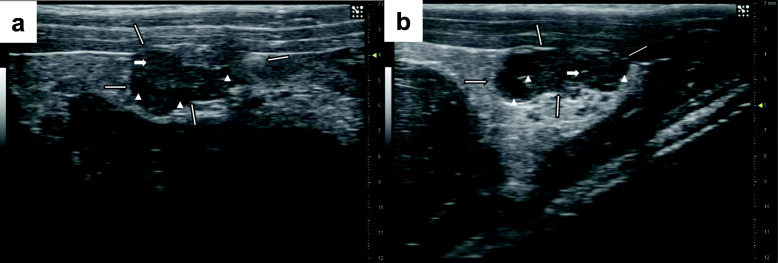
Images of ovarian follicles and corpora lutea in rats using ultrasound biomicroscopy. Legend: Arrows with black outline define the external boundary of the ovary; Arrow-heads indicate follicles; White arrows indicate corpora lutea

All UBM cine-loops were analyzed by a single analyst (JI), who was blinded to the animal treatment group, using Imagyne© (Synergyne Imaging Technology, Inc., Saskatoon, Saskatchewan, Canada) software. The scale used by Imagyne© was set from the scale in the cine-loop. Each cine-loop was evaluated at full and half speed at different times. The number of follicles present at different locations was estimated as the ultrasound transducer was swept across the ovaries from medial to lateral aspect. Follicles greater than 400 μm were enumerated and recorded. Follicle size was assessed roughly using the scale on the side of the image; if follicles close to the criteria for size were observed, a screen shot of the image was recorded and the diameter of the follicle was measured and recorded. A screenshot image was acquired at the maximal area of the ovary in the plane imaged for each cine-loop (Fig. 1). The size of each ovary at its maximum dimension was measured by drawing a line across the largest dimension of the ovary and recording the length of that line. The outline of the ovary was traced and the total area of the identified region was recorded. The number of new corpora lutea were enumerated in screenshots of ovaries taken at the maximum dimension on study days 25, 29, 35 and 39. The newest corpora lutea were measured using a line measurement for the longest and widest dimension. The outside boundaries of the newest corpora lutea were traced and the area measurement recorded. All corpora lutea observed in the ovaries on each of these days were also counted. The right and left ovarian volumes and corpora lutea area were averaged and the number of follicles (≥ 400 μm) and corpora lutea in both ovaries were summed for analyses.

### Serum Estradiol

Serum 17β-estradiol was measured in duplicate using radioimmunoassay (MP Biomedicals, Solon, OH). Assay limits were 10–3000 pg/mL and the intra-assay variation was 9%.

### Statistical analyses

Analyses were performed using R Statistical Software (version 4.0.0; R Foundation for Statistical Computing, Vienna, Austria). Data were assessed for normality and outliers prior to analyses. Differences between study groups were assessed with the nlme package [26] (body weight at pellet insertion and sacrifice, uterus and ovary weight, days/cycle phase variables), with animal ID as a random variable, or chi-square [27] (cycle type classification variables). Body weight and food consumption were analyzed as repeated measures using the nlme package. Treatment group, time, and group*time interaction terms were included in the model as fixed effects and animal ID as a random effect. Estradiol concentration, ovarian volume, and corpus luteum area were analyzed as repeated measures using the nlme package with estrus phase (categorical variable) included as a changing covariate and animal ID as a random effect. Follicle and corpora lutea counts were analyzed as repeated measures using a Poisson model with the lme4 package [28] with estrus phase (categorical variable) included as a changing covariate and animal ID as a random effect. Significance was set at *p* < 0.05 and data are presented as mean ± standard deviation.

## Results

**Fig. 2:**
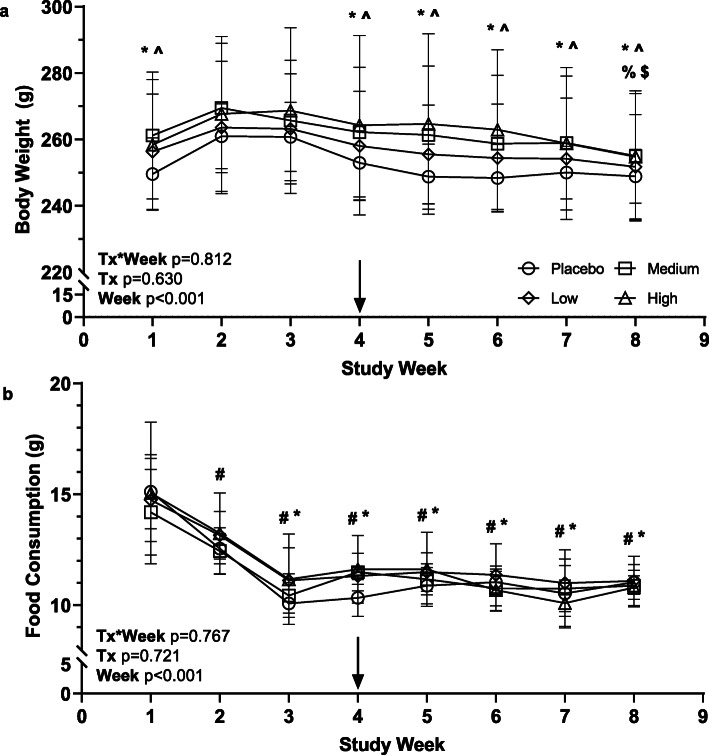
Body weight (**a**) and food consumption (**b**) varied with time across the study. Legend: Arrow = day of pellet insertion, # = different from week 1, * = different from week 2, ^ = different from week 3, % = different from week 4, $ = different from week 5

Body weights (Fig. 2a) and food consumption (Fig. 2b) of all animals varied over time (*p* < 0.001), but not by treatment group (*p* > 0.5), nor was there a treatment group*study week interaction (*p* > 0.7). Animals initially gained weight (~ 9 g) from week 1 to week 2 and remained weight stable from week 2 to week 3. Animals lost weight (~ 5 g) from week 3 to week 4 and remained weight stable until week 8. All animals initially ate more food per day (~ 15 g) during weeks 1 and 2 but slowly stabilized to 10-11 g/day by week 3. The 10–11 g/day consumption volume was maintained until the end of the study. The placebo, low, medium, and high dose groups did not differ with regard to body weight (Table 1) on the day of pellet insertion or on day of termination (*p* > 0.8). Weights of the ovaries and uterus taken at termination did not differ among treatment groups (*p* > 0.2; Table 1).

**Table 1:** Animal body weight and organ weights at pellet insertion or termination

	Placebo	Low	Medium	High	*p*-value
Body weight at Pellet Insertion (g)	253.5 ± 11.8	257.8 ± 17.8	261.0 ± 19.9	263.9 ± 27.8	0.944
Body weight at Termination (g)	248.6 ± 8.1	251.6 ± 15.9	253.9 ± 20.4	255.5 ± 18.6	0.858
Ovary weight (g)	0.11 ± 0.02	0.12 ± 0.02	0.12 ± 0.02	0.11 ± 0.01	0.310
Uterus weight (g)	0.55 ± 0.11	0.52 ± 0.15	0.52 ± 0.14	0.42 ± 0.14	0.236

Mean ± SD

### Estrus cycle

**Fig. 3:**
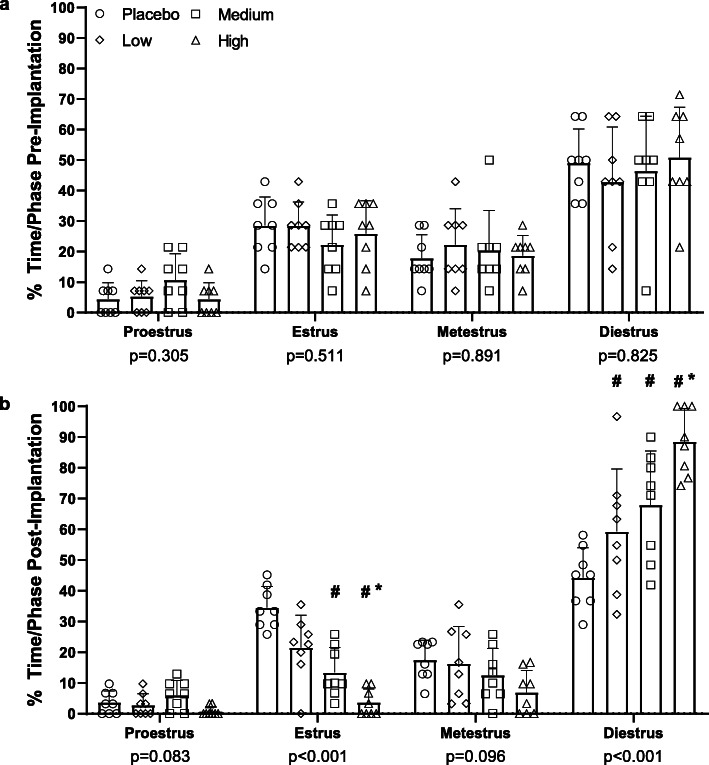
Proportion of time spent in a specific cycle phase prior to (**a**) or following pellet insertion (**b**). Legend: # = different from Placebo dose within cycle phase, * = different from Low dose within cycle phase

**Fig. 4:**
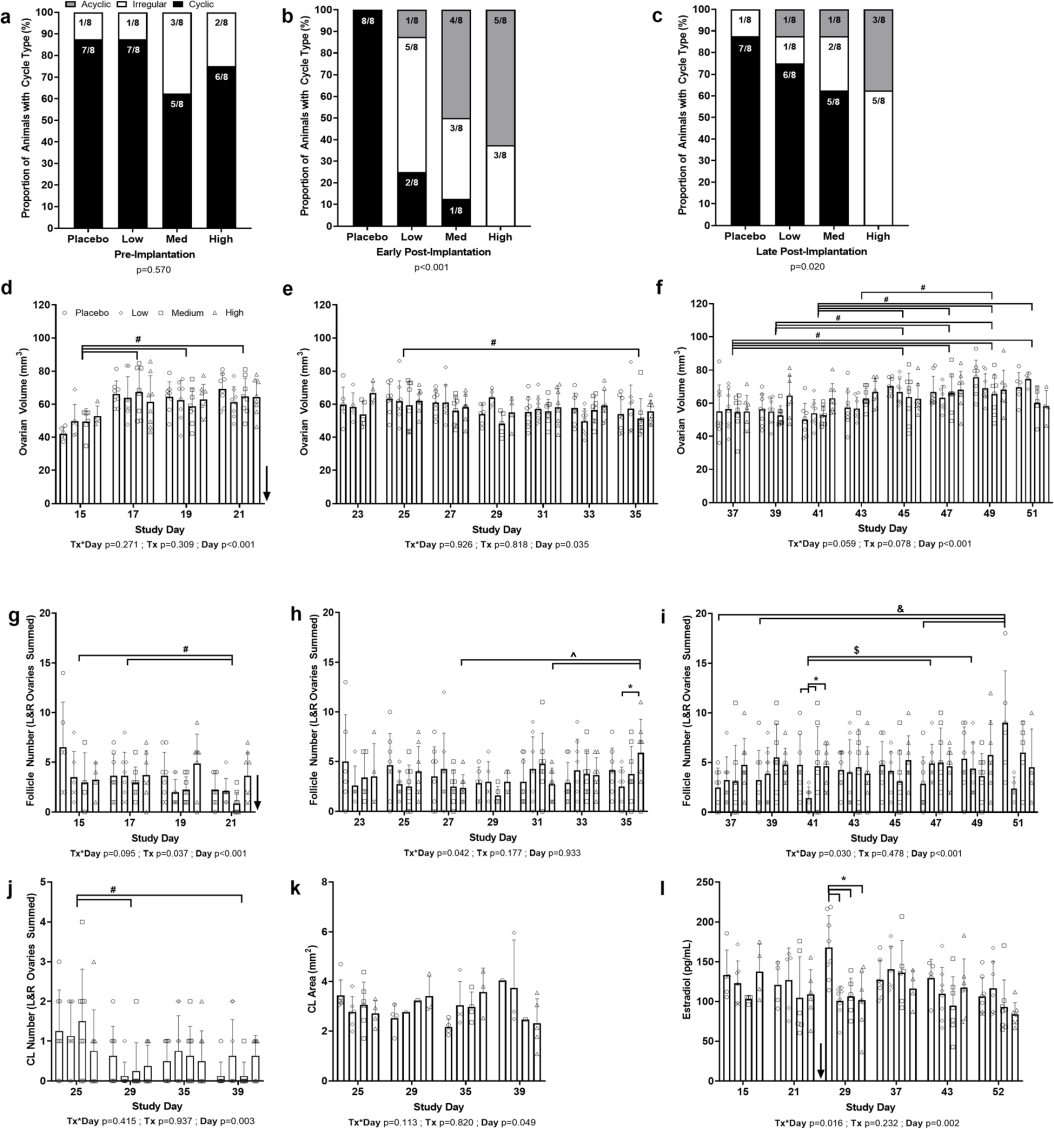
Cycle type classifications (**a**-**c**), ultrasound biomicrocope measurements (**d**-**k**), and estradiol concentrations (**l**). Legend: Arrow = day of pellet insertion, lines with # = difference between days (all treatment groups combined), lines with * = difference between treatment groups within one day, lines with & = difference between study days within the placebo group, lines with $ = difference between study days within the low dose group, lines with ^ = difference between study days within the high dose group

Prior to pellet insertion, animals in each treatment group spent similar total amounts of time in each of the phases of the estrus cycle (Fig. 3a; *p* > 0.150). The proportion of animals classified as cyclic and irregular were similar among the four treatment groups prior to pellet insertion (Fig. 4a; *p* = 0.570); no animals classified as acyclic. Following pellet insertion, treatment groups varied in the total amount of time spent in diestrus and estrus (Fig. 3b; *p* < 0.001) but not in proestrus and metestrus (*p* > 0.050). There was a dose-dependent increase in the total number of days spent in the diestrus phase and reduction in total number of days spent in the estrus phase, with animals in the high dose group spending the greatest number of days in diestrus and fewest number of days in estrus than both the placebo and low dose groups. The medium dose group spent more days in diestrus and fewer days in estrus than the placebo group. The low dose group spent more days in diestrus than the placebo group. In the first two weeks following the pellet implantation (early post-implantation), there was a dose-dependent shift in the proportion of animals classified as cyclic, irregular, and acyclic (Fig. 4b; *p* < 0.001). There were no cyclic animals observed in the high dose group and few animals classified as cyclic in the low and medium dose groups. In the second two weeks after pellet implantation (late post-implantation), the dose-dependent shift in the proportion of animals classified as cyclic, irregular, and acyclic continued (Fig. 4c; *p* = 0.020). There were no cyclic animals observed in the high dose group. In the medium dose group, fewer animals were classified as acyclic and more animals were classified as cyclic than in the high dose group. In the low dose group, fewer animals were classified as irregular and more classified as cyclic than in the medium dose group.

### Ultrasound biomicroscopy

Ovarian volume assessed in the week prior to pellet insertion (Fig. 4d) varied by study day (*p* < 0.001) with volumes on day 15 being lower than days 17, 19, and 21 (*p* < 0.001). Ovarian volume assessed early post-implantation (Fig. 4e) and late post-implantation (Fig. 4f) varied by study day (*p* < 0.040). Early post-implantation ovarian volumes averaged across groups on day 25 were greater than on day 35 (*p* = 0.022). Late post-implantation day 37 ovarian volumes were lower than days 45, 47, 49, and 51 volumes (*p* < 0.020), day 39 ovarian volumes were lower than days 45, 47, and 49 volumes (*p* < 0.008), day 41 ovarian volumes were lower than days 45, 47, 49, and 51 volumes (*p* < 0.020), and day 43 ovarian volumes were lower than day 49 volumes (*p* = 0.011).

The number of follicles with a diameter greater than 400 μm in the pre-implantation time period (Fig. 4g) varied by study day and treatment group (*p* < 0.040). Post-hoc analyses did not reveal significant differences among treatment groups. There were more large follicles on days 15 and 17 than on day 21 (*p* < 0.003). A treatment group*study day interaction was observed for follicle number in the early post-implantation period (Fig. 4h; *p* = 0.042), such that on day 35 there were fewer follicles in the low dose group compared with the high dose group (*p* = 0.038). Additionally, there were more follicles on day 35 than on days 27 and 31 (*p* < 0.050) in the high dose group. A treatment group*study day interaction (*p* = 0.030) was observed for follicle number in the late post-implantation period (Fig. 4i). On study day 41, the low dose group had fewer follicles than the placebo, medium, and high dose groups (p < 0.020). The low dose group had fewer follicles than the placebo dose group (*p* = 0.005) on study day 51. For the placebo group, follicle number on day 51 was greater than on days 37, 39, and 47 (*p* < 0.004). For the low dose group, follicle number on day 41 was lower than on days 47 and 49 (*p* < 0.040).

The number of corpora lutea from recent ovulations (Fig. 4j) differed by study day (*p* = 0.003). Fewer new corpora lutea were observed further from pellet implantation (days 29 and 39) compared to closer to pellet implantation (day 25; *p* < 0.006). The area of the new corpora lutea (Fig. 4k) differed by day (*p* = 0.049); however, post-hoc analyses did not reveal significant differences for specific days or among groups.

### Estradiol

A treatment group*study day interaction was observed for serum estradiol concentration (Fig. 4l; *p* = 0.016). In the blood sample taken 7 days (day 1 of week 5) after the insertion of the ENG pellets, all of the low, medium, and high dose groups’ estradiol was lower than the placebo group (*p* < 0.007).

## Discussion

The number of women worldwide choosing subdermal rod and IUD LARC modalities as their HC option is increasing due to the reduced need for user compliance, lower steroid dose, and long-term protection. However, our understanding of the impact of HC therapy on non-reproductive body systems is incomplete or unknown. Determining the mechanistic impact of LARC modality use on non-reproductive organ systems can be accelerated if a validated rodent model is available. We determined high, medium, and low doses of ENG for reproductively mature rodents, based on published average ENG release rates from available subdermal rod modalities at years 1, 2, and 3 of use in women [3, 13], and tracked estrus cyclicity and functional ovarian data to test how well this rodent model simulated human response to subdermal rod LARC modalities.

We observed dose-dependent differences in estrus cyclicity using daily assessments of vaginal cytology across the study. The most effective suppression was observed for the high dose implant group. A larger proportion of acyclic and irregular cycles was observed in animals in the high dose group in the early and late post-implantation periods. There was a dose-dependent impact on the time spent in diestrus and estrus (Fig. 3b) following pellet insertion; the high-dose group spent the most time in diestrus and the least time in estrus. The low dose did not impact estrus cyclicity for as long a duration as did the medium or high doses; however, a disruptive effect on the estrus cycle was observed early following pellet implantation.

Over the first year of use of an ENG subdermal rod LARC, progressively more women experience amenorrhea (no menstrual bleeding for 90 days or longer), with the proportion of women experiencing infrequent, frequent, prolonged, or irregular bleeding patterns remaining consistent or decreasing across the same time frame [29, 30]. Over the next years of use, the proportion of women experiencing amenorrhea begins to decrease and the number of women experiencing normal menstrual cycle patterns increases slowly [30]. In a three-year trial of subdermal rod LARC use, no women reported consistent amenorrhea for the entire three years of use [13]. The suppressive effect of the ENG subdermal implant on the ovaries is overcome slowly in conjunction with the decrease in the dose release rate over time in women. We observed similar responses over time in our rodent estrus cycle monitoring [13, 29, 30]. Though menstrual cycling occurs in women using subdermal rod LARC modalities, ovulation does not typically occur until the third year of use [13]. The dose received in year 3 of human use is equivalent to our low dose ENG implant, where we observe the greatest number of animals with normal estrus cycles in the late post-implant period. The observed estrus patterns across the three doses of ENG utilized in the present study were like the patterns of menstrual cyclicity reported in women over each of the three years of subdermal rod LARC use [29, 30].

A strength of the current investigation is the frequent assessment of ovarian function using UBM. We interpreted the UBM data to mean that the implants did not disrupt what was currently happening in the ovaries, as occurs with subdermal rod insertion in humans, but resulted in suspension of follicle atresia and corpus luteum regression. In the pre-implantation phase of our study, UBM ovarian volume and follicle number data were consistent with the estrus cycle data. No differences were observed among treatment groups and the anticipated normal ovarian dynamics were observed. In the early post-implant phase, ovarian volume appeared to enter a holding pattern and the follicle number data did not reflect a normal cycling structure, with atresia appearing to be paused. UBM ovarian volume and follicle number data in the late post-implant phase reflected a rebound from the interruption in ovarian dynamics observed in the previous two weeks. In a 12-month study of ENG subdermal rod LARC in women, there were two types of ovarian activity noted: 1) follicles not growing above 10 mm (dominant follicle selection not observed) and 2) follicles growing above 10 mm (dominant follicles selected) but with no luteal structures or activity observed [4]. Menstrual status did not completely reflect ovarian function [4]. Amenorrhea, reduced menstrual flow, and frequent menstrual bleeding were reported.

In a later comparison of ENG and LNG LARC options, more anovulatory follicular cysts were documented in the LNG users [13]. In an earlier study using 8- to 12-week-old Wistar rats, ENG subdermal rods (Implanon, Organon, Oss, Netherlands) were cut to match animal weight and implanted for 50 days. No differences were observed in antral follicle count; however, more deformed enlarged follicles, as assessed by histology, were observed in the contraceptive groups [17]. Caution should be taken in examining the responses to the ENG subdermal rod. The size of the inserted implant was adjusted based on animal weight; however, the daily hormone release rate from the implanted portion will not have been reduced and information regarding the impact on the estrus cycle were not provided. Though UBM data from the present study did not show the expected dose-dependent response, our ovarian data strongly support disruption of ovarian function with all doses of the ENG implant used in the study.

We observed that corpora lutea were not regressing normally following pellet insertion. Corpora lutea structures are evident in UBM scans but the function of the luteal structures in the ENG groups corpora lutea was likely different from that of the control group. The short inter-ovulatory interval in the rat and the slow rate of luteal regression mean the luteal structures from several previous estrus cycles are present in the ovaries concurrently. In the present study we endeavored to quantitate only the most recent corpora lutea in an attempt to use the model to best mimic the human condition. Though the publication has since been retracted due to discovery of incorrect data in some case report forms [31], four of 131 cycles (28 days of use) of ENG implant use in the first 3 years of implantation had recorded ovulations in the final year (months 30–36) [32]. Similarly, in a comparison of the ENG and LNG subdermal rod implants in humans, ovulation was reported in ENG and LNG subdermal rod groups. Elevated progesterone concentrations, interpreted to mean development of luteal structures, were reported in the ENG subdermal rod group in months 30 and 33, respectively, and in months 12, 18, 30, 33, and 36 in the LNG subdermal rod group [13].

Estradiol concentrations in animals in the present study showed a treatment group*time interaction. In the first assessment following implant insertion, all treatment groups had suppressed estradiol compared to the placebo group; subsequent assessments were no longer different. Data from the present study are similar to human reports where follicular development and estradiol concentrations were initially suppressed, then ovarian activity slowly increased after 6 months of use and FSH and estradiol concentrations gradually rose and were compatible with follicular growth observed using ultrasonography [13].

No changes in animal grooming or ambulatory activity were observed throughout the study. Animals in all groups exhibited similar changes in weight and eating behavior over time, independent of ENG dose. The differences we observed across time were attributed to the initial stress of single housing (week 0) and change to a phytoestrogen-free diet (week 1) which initially increased food consumption then subsequently stabilized (week 3). Animals did not differ in weight and food consumption remained stable from the time of randomization and implant insertion (week 4) to the end of the study. In humans weight gain, whether perceived or physiological, is reported as one of the most significant adverse events leading to discontinuation of study protocols in clinical trials. Subdermal rod implant users were more likely to report weight increases than participants in copper IUD control groups [33]. In a cohort of healthy women (30–55 years old at study entry), weight gain over two consecutive 4-year periods was approximately 2 ± 5 kg/period [34], which is similar to reports of weight change in ENG and LNG LARC implant trials [33, 35–38]. Reports indicating increases in weight with LARC use found significant predictors of weight gain to be age over 35 [35], height over 150 cm [35], country of participation [35] and ethnicity [38]. In a study where ENG subcutaneous rods (Implanon, Organon, Oss, Netherlands) were sized according to body weight of Wistar rats, animals in the treatment groups weighed more than the control animals [17]. Dosing concentration and choice of implant in the present study influenced food intake and body weight in a manner similar to that observed in human clinical trials and support the use of this model for assessing impacts on non-reproductive organ systems in future investigations.

UBM has been used in short term to visualize reproductive tissues in rodents [24, 25, 39, 40] and has provided the opportunity to utilize imaging technology in long-term studies in rodents. Strengths of the present study are that it is the first study to utilize UBM to examine ovarian changes frequently and over an extended period of time (35 days) in association with daily estrus monitoring. UBM provides dynamic information over time in multiple animals, in contrast to the single time points measured with histological studies. We were unable to assess progesterone and ENG concentrations, in addition to serum estradiol, with the small sample volumes (< 200 μL) we were able to collect from animals each week. To ensure animal and ultrasonographer health, we chose to limit isoflurane exposure to alternate days; however, we believe this scanning schedule masked our ability to observe some of the changes we anticipated. Thus, we included both serum estradiol assessments and UBM measures as corroboration of ovarian function for our estrus cycle monitoring.

## Conclusions

We successfully developed and validated an animal model of implantable ENG LARC at daily dosing rates that are physiologically equivalent to the average dosing rates experienced by human ENG LARC users during the first three years of use. Follicles and corpora lutea present at ENG pellet implantation or that emerged following ENG pellet implantation enter a state of stasis. Other than the expected impacts on ovarian function and estrus cycling reported, no side effects were observed in the animals in the study. We have shown that the doses calculated independently demonstrated similar impacts on estrus cyclicity to those menstrual cyclicity changes reported in humans during each of the three years of use. Each of the doses can be used independently or with each other in a sequential manner over time, depending on the hypothesis being tested by the investigator. The new model can be used to assess cellular and whole tissue impacts of ENG LARC on non-reproductive organ systems in future investigations to elucidate the broad systemic impact of this widely used medication.

## Data Availability

The datasets generated and analysed during the current study are not publicly available but are available with appropriate approvals from the NASA Life Sciences Data Archive.

## Abbreviations

ENG: Etonogestrel
HC: Hormonal contraception
UBM: Ultrasound biomicroscope
LARC: Long-acting, reversible contraception
IUD: Intra-uterine device
FDA: Food and Drug Administration
LNG: Levonorgestrel
BMD: Bone mineral density
Tx: Treatment group
